# Literacy Toy for Enhancement Phonological Awareness: A Longitudinal Study

**DOI:** 10.1007/978-3-030-58805-2_44

**Published:** 2020-08-12

**Authors:** Carlos Ramos-Galarza, Hugo Arias-Flores, Omar Cóndor-Herrera, Janio Jadán-Guerrero

**Affiliations:** 8 Institute Integriert Studieren, JKU Linz, Linz, Austria; 9 Jack Baskin School of Engineering, UC Santa Cruz, Santa Cruz, CA USA; 10 Dipartimento di Meccanica, Politecnico di Milano, Milan, Italy; 11 Support Centre for Students with Special Needs, Masaryk University Brno, Brno, Czech Republic; 12 Facultad de Psicología, Pontificia Universidad Católica del Ecuador, Av. 12 de Octubre y Roca, Quito, Ecuador; 13 Centro de Investigación en Mecatrónica y Sistemas Interactivos MIST/Carrera de Ingeniería en Ciencias de la Computación/Maestría en Educación mención Innovación y Liderazgo Educativo/Carrera de Psicología/Carrera de Administración, Universidad Tecnológica Indoamérica, Av. Machala y Sabanilla, Quito, Ecuador

**Keywords:** Phonological awareness, Literacy toy, Inclusive toys, RFID, PECFO

## Abstract

In this report it is presented the results of a longitudinal pre-experimental study, it was realized a technological intervention to stimulate the phonological awareness through a tangible reading toy based on the RFID technology, consisting of a teddy bear and 30 letters in 3D from the Spanish alphabet. This study started with a sample of 200 children, from them, there were selected 17 children aged between 6 and 7 years (*M*_*age*_ = 6.47, *SD* = .51) with a phonological disorder from an educative institution. The procedure consisted of obtaining pre-test and post-test values with the Evaluation of Phonological Awareness (PECFO). Sampling inclusion criteria considered children presenting problems of phonemes’ recognition and its relationship with graphemes. During 30 weeks it was realized an intervention with the technological toy and at the end of the sessions, it was applied the post-test. Results of phonological awareness showed statically significant differences among the pre (*M* = 12.88, *SD* = 3.53) and post-test (*M* = 17.17, *SD* = 2.96) this contributes to the empirical evidence of the intervened group improvement in this cognitive function *t*_*(16)*_ = −3.67, *p* = .002. From this research it is projected proposing technological innovations contributing in the treatment of children’s cognitive difficulties.

## Introduction

Cognitive development is a central issue in the context of technological innovations for children’s disorders treatment. Because of it, in this article, it is reported a longitudinal pre-experimental study, where it was realized an interactive device for children to improve their phonological development. Following, the benefits of technology usage in the treatment of cognitive difficulties are described, as well as the proposal of a reading tangible toy, as technological innovation, for the treatment of phonological awareness’ difficulties. Afterward, it is explained the longitudinal pre-experimental study carried out.

### Benefits of Technological Usage for Cognitive Issues

The use of ICT inserted in the educative field allows generating new forms of production, representation, diffusion and knowledge accessibility [1], which represents a constant innovation for education.

The use of a variety of devices and technological resources, adding modern students’ innate abilities for using them, makes possible offering educative interactive innovations, different to those accustomed [2], which allows students to enjoy the learning process, generating on them an inner motivation, these emotions influence not just in motivation but improving significantly learning experience and academic achievements [3].

### Technological Devices Used in Phonological Awareness and Cognitive Difficulties

Plenty studies are approaching technological usage to work on cognitive difficulties and phonological awareness, an example of it is the Project called Petit Ubinding, that has been designed by the University of Barcelona [4], which measured the impact of an educative method to stimulate reading in children belonging to the first grade of primary school, including on-line learning sessions. Another study conducted in a sample of deaf students about reading, orthography, and phonological abilities found that technological usage allows the improvement of these abilities [5]. On the other hand, the usage of computer-assisted pronunciation training (CAPT) is used in learning a second language for a variety range of age and technological use supports children identifying phonemes and bad-word pronouncing, suggesting to users the option to improve these errors [6]. There are other alphabetization programs based on the web offered possibilities, which have reported positive evidence improving phonological abilities that were worked on, such as phoneme-grapheme correspondence, segmentation of phonemes and word fluency [7].

Another field in this same research line is the usage of toys and inclusive games for children with disabilities rehabilitation, and in the same manner, these could be used in children presenting learning disorders such as dyslexia, dysgraphia, and dyslalia, reflecting excellent results in the learning process [8, 9]. The previous investigation about the usage of web extension assisting reading and writing problems reported significant results of treating these issues, as well as increasing motivation and child’s working frequency [10].

Implementing the usage of technological tools is substantive in children’s initial reading process, as it is evidenced in the longitudinal study of early reading development through technological media. Intervened children with this type of stimulation improve the automatic integration of letters and sounds and a variety of measures that assess early reading and language abilities [20].

### The Necessity to Create a Friendly Device for Children with Cognitive Difficulties

Children must be prepared to acquire abilities and knowledge in the actual world and the application of technology in the learning environment will allow them to use their thinking abilities to reach social and emotional development [11, 12]. Learning environments enriched with technology in its variety [13, 14] will improve attention levels, motivation, knowledge and students’ abilities in a positive way, especially in children with language disorders and special education [15, 16].

### Technological Proposal to Work with Phonologic Awareness

The proposal of a tangible reading toy is based on one of the already mentioned literature, in which it was developed a literacy kit called Kiteracy (Kit for Literacy) to generate interaction of children with Down’s Syndrome in the learning process. The study realized was based on a qualitative technique, using recorded sessions in video with twelve children with Down’s Syndrome belonging to an institute from Spain. This technological proposal is presented in three interactive ways: cardboard cards, a tablet and a technological radiofrequency (RFID) toy and tangible objects.

The task was conducted by special education teachers and 12 children presenting Down’s Syndrome. There were design three experimental sessions with every child consisting of pair-work (professor-child). From these sessions, it was possible identifying that tangible interaction offers an enjoyable moment for children. Surveys and interviews’ data collected from the information given by professors revealed that tangible objects offered higher adaptability to create reading pedagogic strategies [21].

**Fig. 1.**
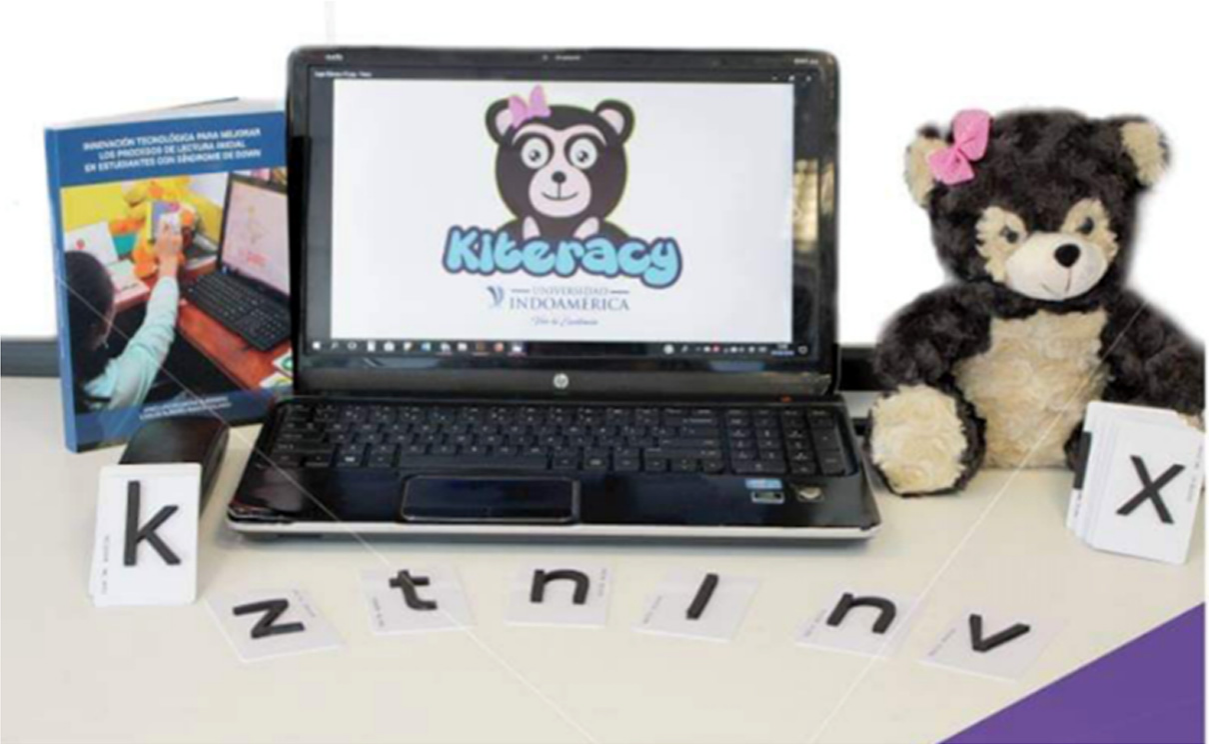
Tangible reading toy Kiteracy-Pifo.

Taking into consideration this experience and with positive results in the interaction, a new research question appeared, “It is possible to use the kit to stimulate phonological awareness in children with non-special conditions”, to do it, it was necessary to make a kit´s adaptation to Ecuadorian context, creating a kit called Kiteracy-PiFo (Fig. 1) based on the Picto-Phonic (PiFo) alphabetization methodology, which is composed by a teddy bear with an RFID lector incorporated and 30 labels representing alphabet´s letters. There were manufactured 25 kits to conduct the pre-experimental longitudinal study, to assess the effectiveness of interventions in children aged between 6 and 7 years [22].

The teddy bear contained cards RFID with the technical specification LANMU Smart ID Card Reader EM4099 USB – Proximity Sensor of 125 kHz. Also, there were used 30 cardboards made of plastic RFID with letters in pieces made of foaming material. The software allows the association between the card´s coding and phonemes sound, the visualization of the grapheme and the interactive video of trace and vocalization.

## Method

### Research Design

There was applied a pre-experimental longitudinal study since it was worked with a group of students presenting alterations in the phonological awareness.

### Participants

The sample started with 200 students, from them, 17 children aged between 6 and 7 years with phonological alterations were selected (Mage = 6.47, SD = .51). According to gender, 6 (35.3%) were females and 11 (64.7%) were males. These children belonged to the educative private system of Quito- Ecuador.

### Instruments

For obtaining pre and post-test values, it was used the phonological awareness evaluation test (PECFO) [17], this test allows evaluating phonological awareness development in children.

### Procedure

The pre-experimental longitudinal study was conducted in an educative institution. It started with the pre-test phonological awareness test. Afterward, it was realized a technological intervention with the device to improve phonological awareness for 30 weeks. Finally, in the post-test, the impact of this technological intervention was analyzed.

It is important to highlight that this research was approved by the Ethical Committee of Investigation with human beings of the University Indoamerica of Ecuador. Participant’s representatives were asked to sign the informed consent of voluntary participation and children were asked to give their assent by signing in a form as well, of accepting being part of the study. Throughout this research ethical standards of investigation with human beings were followed, protecting participants’ physical and psychological integrity at all times.

### Statistical Analysis

Once the statistical hypotheses were proven, it was conducted a comparison of means with a T-test procedure for related samples between the pre and post-test realized. Also, there were applied statistical central tendency and dispersion measures to characterize the data.

## Results

In the beginning, descriptive values found were analyzed in the different measures. Table 1 shows these values.

**Table 1.** Descriptive results of the variables valued. Note: Mn (minimum), Mx (maximum), M (mean) and SD (Standard Deviation).

*Measure*	*Mn*	*Mx*	*M*	*SD*
Pre-test	4.00	18.00	12.88	3.53
Post-test	11.00	23.00	17.17	2.96

The second analysis realized was the comparison of means between the pre and post-test of the phonological awareness variable. Table 2 shows the results found.

**Table 2.** Comparison realized between the pre and post-test of the linguistical variable valued.

	*M*	*SD*	*Mn*	*Mx*	*T*	*Df*	*Sig.*	*D*
Pre-test vs. Post-test	−4.29	4.82	−6.77	−1.82	−3.67	16	.002	.68

**Fig. 2.**
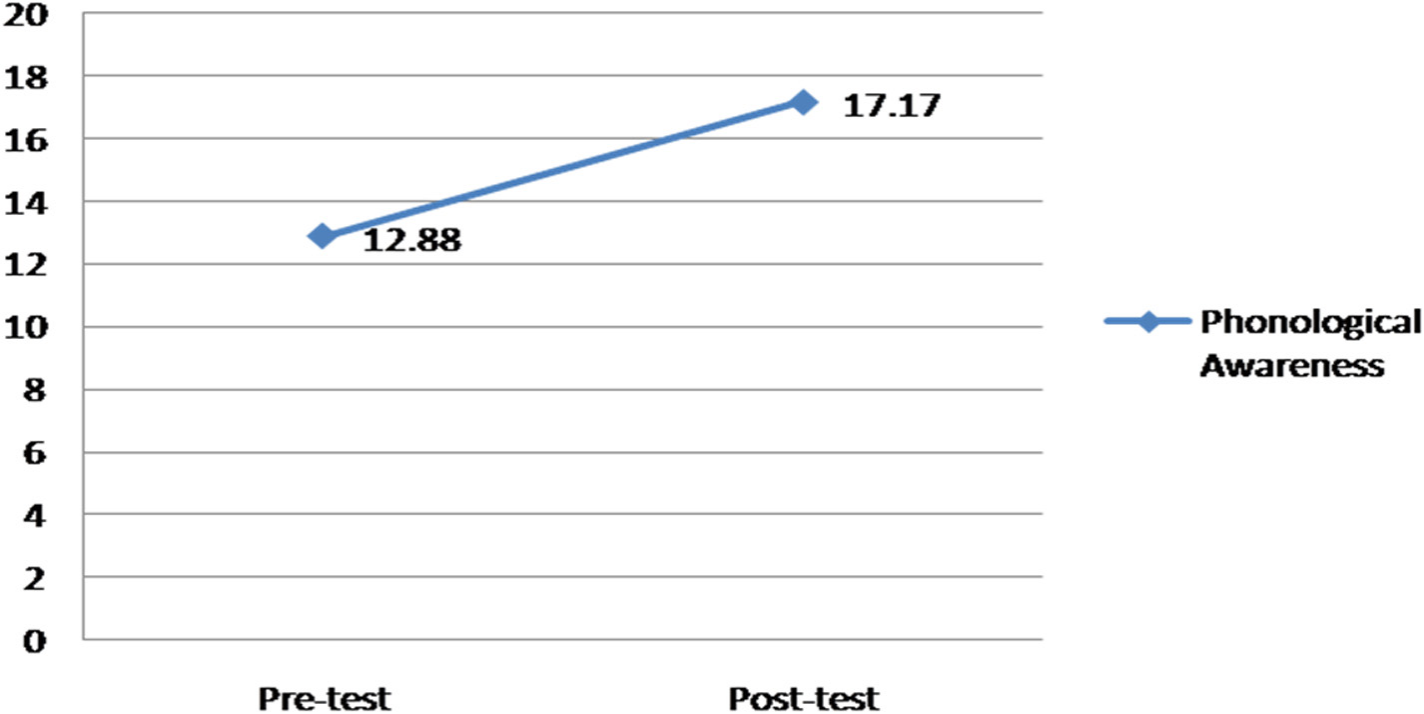
Pre and post-test means differences.

Figure 2 presents phonological awareness means’ differences of pre and post-test.

## Conclusions

This work reports an investigation that analyzed the impact of the technological development of Kiteracy-Pifo to intervene in the phonological awareness difficulties in children aged between 6 and 7 years.

Results found affirmed that its application has positive results since there were found statistically significant improvements in the phonological awareness between the pre and post-test. Results found in this investigation are concordant with the results found in previous research such as the one conducted by Thompson et al. [18] and Gilakjani and Rahimy [19], who have reported that technological use improves the oral phoneme-grapheme decoding precision as well as reading rating, contributing to significant improvement in pronunciation learning; this allows to affirm that children with learning difficulties will be benefited with reading, pronunciation, phonological awareness and writing abilities learning through technological use.

The future investigation that is proposed is related to the creation, production, and implementation of new technological inclusive devices contributing to the treatment of children with cognitive difficulties, as well as the implementation of these devices in usual educative contexts to stimulate cognitive processes.
